# Short Term Effects of Inner Engineering Completion Online Program on Stress and Well-Being Measures

**DOI:** 10.3389/fpsyg.2022.814224

**Published:** 2022-04-27

**Authors:** Preeti Upadhyay, Akshaj Joshi, Isha Mishra, Lauren Kelly, Lena Novack, Sepideh Hariri, Kestutis Kveraga, Balachundhar Subramaniam

**Affiliations:** Beth Israel Deaconess Medical Center, Brookline, MA, United States

**Keywords:** Yoga, meditation, perceived stress, Isha, IECO

## Abstract

**Introduction:**

The Covid-19 pandemic has been a major disruptor of routine life, resulting in increased stress and predisposing people to negative outcomes, such as insomnia, anxiety and hopelessness. Mind-body interventions have improved concentration, emotional balance, and positive emotions, with an enhanced sense of productivity, and self-confidence. We therefore hypothesized that exposure to an online mind-body intervention, “Inner Engineering Completion Online (IECO),” would reduce stress and promote well-being.

**Methods:**

This prospective cohort study enrolled participants registered for the IECO courses, which for the first time were delivered remotely, online. Participants learned a 21-min meditation practice called Shambhavi Mahamudra Kriya during the course, which incorporates controlled breathing and mediation techniques. Each enrolled participant was asked to complete self-reported electronic surveys at three key time points: at the time of consent, immediately after completing IECO, and 6 weeks after IECO completion. Effects of IECO practice were assessed using four well-validated neuropsychological scales: Perceived Stress Scale (PSS), Positive Emotion/Relationship/Engagement Scale (PERMA) Profiler, Pittsburgh Sleep Quality Index (PSQI), and Mindful Attention Awareness Scale (MAAS). A Signed Rank test was used to analyze the survey data and *P*-values of < 0.05 were considered statistically significant.

**Results:**

Of the 375 participants interested in participation, 164 participants were eligible. Sixty-eight participants completed surveys at all time points and were identified as compliant participants. The baseline median score for PSS in compliant participants (*n* = 95) was 13.5 (IQR 9, 18); immediate post-IECO median PSS score was 12 (IQR 8, 16) demonstrating a 1.5 unit decrease in PSS scores (*p*-value = 0.0023). Similarly, comparing PSS scores in compliant participants (*n* = 68) for immediate Post IECO [11.5 (IQR 8, 15.5)] to PSS scores at six weeks [8 (IQR 4.5, 12.5)] showed a statistically significant 3.5-unit decrease, indicating a reduction in stress upon routine practice of the intervention (*p* < 0.0001).

**Conclusion:**

Incorporating the remotely delivered mind-body intervention *Shambhavi Mahamudra Kriya* into daily life via the IECO program over as few as 6 weeks produced a significant stress reduction, improvement in sleep quality and mindfulness.

**Clinical Trial Registration:**

[ClinicalTrials.gov], identifier [NCT04189146].

## Introduction

Stress refers to physiological changes occurring in an organism in response to a potentially adverse change in the environment, whether real or perceived (Joëls and Baram, 2009). This stress response is mediated through an array of neurotransmitters, neuropeptides and steroids, particularly glucocorticoids and catecholamines (Joëls and Baram, 2009; Golbidi et al., 2015). Although the stress response is postulated to allow organisms to adapt to changes in the environment in the short term, its chronic activation is associated with more significant systemic inflammation, a disrupted microbiome, and a greater risk for cardiovascular disease, aside from negative mental health consequences (Golbidi et al., 2015; Househam et al., 2017).

Despite routine use of anxiolytics in treating individuals with high stress or anxiety disorders, a combination of pharmacotherapy and mind-body interventions is being increasingly explored as a means of aiding such patients (Lee et al., 2007). Meditation may be a cheap, relatively easy to learn, and safe means of combating stress and increasing feelings of well-being in daily life (Goyal et al., 2014; Khalsa, 2015; Edwards et al., 2018).

Meditation, a means of developing insight and regulating internal states of consciousness, has been a part of Eastern, Western, and various Indigenous spiritual traditions for millennia, but is only recently undergoing scientific scrutiny in the context of health and disease. “Mindfulness” can be defined as the state of moment-to-moment awareness of present experiences or the meditation method. This state of insight development and internal self-regulation is achieved through attention control, emotion regulation, and contemplation of self, i.e., self-awareness (Tang et al., 2015).

Meditation practices are associated with positive effects on autonomic nervous system activity (Libby et al., 2012; Koerten et al., 2020), brain functionality (Tang et al., 2015), and immune function (Househam et al., 2017). Further, these practices have shown to improve concentration, emotional balance and positive emotions, with an enhanced sense of productivity, and self-confidence (Allen et al., 2021). Negative emotions such as stress, anxiety, and depression reduce with regular practice (Fjorback et al., 2011; Chang, 2021; Upadhyay et al., 2022).

While there are many different meditative traditions and practices, each of which may have differential effects on physiology and health (Tang et al., 2015; Brandmeyer et al., 2019), there are some similarities in the delivery patterns. For example, mind body interventions such as Mindfulness Based Stress Reduction (MBSR), which originated from Buddhist traditions, are similar to Inner Engineering Online (IEO) distilled from ancient yogic traditions. Both practices are multi-component programs that incorporate multiple psychological techniques (e.g., perception and cognitive reappraisals), body movements (modified Hatha Yoga and other body work such as Tai Chi and Qi Kong), and several types of meditation (e.g., body scan, mindful eating, Loving-Kindness meditation).

Inner Engineering Online is a multicomponent program, that engages participants in meditation and yogic practices. It is designed to improve an individual’s physical, mental, and emotional health and is a pre-requisite for enrolling in the Inner Engineering Completion Online (IECO) course (Chang, 2021; Upadhyay et al., 2022). The IECO course is a protocol under the Isha school of yoga and involves learning a 21-min meditation, called *Shambhavi Mahamudra Kriya* (Muralikrishnan et al., 2012; Peterson et al., 2017). This meditation practice involves controlled breathing techniques (Pranayama) and mantra chanting (AUM chanting), followed by breath watching meditation.

This study investigates the effects of the IECO program on stress and sleep quality, hypothesizing that the former will decrease, and the latter will increase with regular practice. It is important to note that the absence of stress does not guarantee the presence of well-being. Therefore, the study also investigates whether participants would experience improvements in various aspects of overall well-being, specifically positive emotions, engagement in work and relationships, and the presence of meaning in one’s activities.

Inner Engineering Completion used to be an in-person program like the Mindfulness-Based Stress Reduction format before the SARS-COV-2 outbreak, and often required travel or time off from work. It has been noted that vacations have an ameliorating effect on stress, and that this may be a part of the positive effects of meditation retreats (Epel et al., 2016; Blasche et al., 2021). Therefore, studying the IECO program, which is incorporated into daily life without traveling or taking time off from work is a unique opportunity to test the efficacy of online delivery of a meditation program, and distinguish the effects of the course from any “vacation effect.”

## Materials and Methods

### Participants and Procedure

This observational study was designed to collect longitudinal data from novice meditators who were introduced to “Inner Engineering” as an intervention and followed up for 1 year. Any individual who could read and understand English and showed an interest in completing the IECO program by enrolling for the course with the Isha foundation, could participate in the study. Exclusion criteria included participants below 18 years of age and non-US resident.

Recruitment was done by sharing the study’s REDCap survey link via advertisement emails and participants self-reported on the eligibility criteria (REDCap is an online electronic data capture database).

**FIGURE 1:**
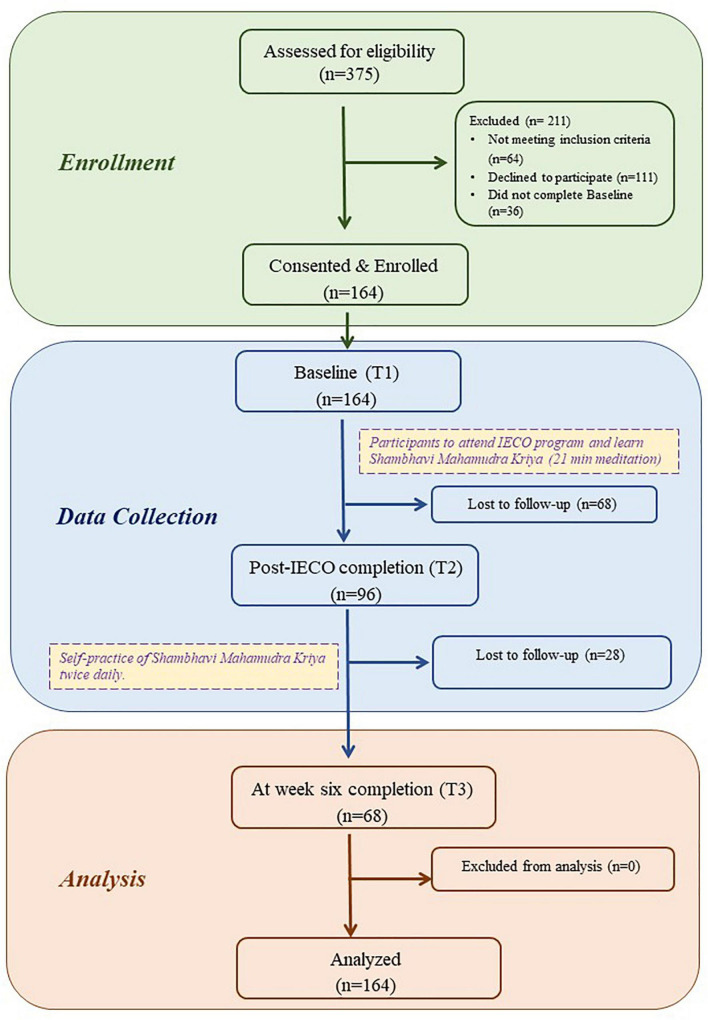
Consort diagram.

Upon consent, participants were asked to respond to the remotely administered survey at the following time points: at Baseline (T1), upon IECO completion (T2), and 6 weeks after IECO completion (T3). These surveys included validated questionnaires assessing stress [Perceived Stress Score (PSS)], sleep quality [Pittsburgh Sleep Quality Index (PSQI)], mindfulness [Mindful Attention Awareness Scale (MAAS)-short scale], and the Positive Emotion/Relationship/Engagement Scale (PERMA) profiler scale, which comprises the following well-being indicators: positive and negative emotions, engagement, relationship, meaning, accomplishment, and health (Figure 1).

Self-reported medical history and any history of addiction or substance abuse (such as alcohol consumption/smoking) along with a 2-min, weekly compliance survey between T2 and T3 were also included. The compliance survey collected information on the frequency and duration of the prescribed practices.

### Intervention

The Inner Engineering training has two components:

1. Inner Engineering Online
2. Inner Engineering Completion Online

Inner Engineering Online is a 4-week self-paced multicomponent program available online. IEO consists of seven online lessons (90-min per session) which involves engaging in intellectual inquiry (resulting in cognitive reappraisal), generating positive emotions, learning Upa Yoga (preparatory Hatha Yoga involving body movement and breathwork), and activation of inner energy (sound and postural yoga) (Sadhguru, 2016). This intervention is a pre-requisite to participate in the IECO program.

Inner Engineering Completion Online involves learning Shambhavi Mahamudra Kriya. This practice is performed for 21 min daily and includes multiple controlled breathing techniques (Pranayama), Aum chanting, and the engagement of bandhas (muscular locks in the abdomen and pelvic floor) applied for 15 min. The practice concludes with about 5 min of breath-watching meditation (Peterson et al., 2017).

### Outcome Measures

#### Stress

Perceived stress was measured by the 10-item Perceived Stress Scale (PSS) (Cohen et al., 1983). Each item was coded as 0 “never” to 4 “very often.” The PSS score can range from 0 to 40. The PSS is the most common measure used in studies of stress, with well-established reliability and validity. It is quite brief and easy to administer online. The reliability of PSS across the duration of the study was 0.89 at Baseline; 0.91 at post-IECO and 0.92 at week six.

#### Mindfulness

Mindfulness was measured using the five-item MAAS (Brown and Ryan, 2003). Each item is coded 1 “never” to 5 “all of the time.” The MAAS is one of the most frequently used mindfulness scale in occupational settings and in general population (Lomas et al., 2019). The reliability of MAAS- short scale was 0.89 at Baseline; 0.92 post-IECO, and 0.90 at week six.

#### Positive Emotion/Engagement/Relationships/Meaning/Accomplishment

Positive Emotion/Engagement/Relationships/Meaning/Accomplishment (PERMA) is a 23-item measure that assesses well-being across five domains (positive emotion, engagement, relationships, meaning, accomplishment). PERMA-Profiler was specifically designed by Butler and Kern (2016) following extensive theoretical and empirical process, to assess well-being across multiple domains. The 15 main PERMA items were chosen through a series of psychometric tests. Eight additional items—one overall well-being item, three negative emotion items, three physical health items, and one loneliness items—were added to the measure, creating the final 23 item measure (Butler and Kern, 2016). Questions are reported on an 11-point scale ranging from 0 to 10, with the endpoints labeled. Scores are calculated as the average of each factor’s items while loneliness is reported as a separate item.

#### Pittsburgh Sleep Quality Index

The PSQI is a 24-item scale that measures sleep disturbances along seven dimensions: subjective sleep quality, sleep latency, sleep duration, habitual sleep efficiency, sleep disturbances, use of sleep medication, and daytime dysfunction. Scores from these seven areas are added together into a global score. Responses are based on most days (and nights) of the previous month (Buysse et al., 1989). The reliability of PSQI global score across study duration was 0.55 at Baseline; 0.44 at post-IECO and 0.61 at week six.

#### Compliance

Compliance was defined as 60% of activity completion during intervention period. This information is collected via the self-reported weekly compliance diaries, collected from the participants.

### Statistical Analysis

In this analysis, descriptive statistics of the data are presented based on variable type and distribution. Continuous data were presented as medians [interquartile range (IQR)] after confirming with the Shapiro-Wilk test that the data did not follow a normal distribution. Differences between different time points were assessed with a Wilcoxon Rank-Sum test. Categorical data are presented as frequencies and proportions and differences between groups were evaluated with chi-square or Fisher’s Exact test, as appropriate. Missing components of PSQI scores were imputed using the median value of all non-missing features. Change in the score on the perceived stress scale was the primary variable of the study. Trial feasibility, compliance, and other outcomes (e.g., sleep quality, mindfulness, and other well-being measures) were defined as the secondary outcomes. Generalized estimating equations (GEE) were performed to test whether time predicted PSS, MAAS, and PSQI scores. In *post-hoc* analyses, we also assessed differences in scores for those participants who dropped out of the study at the post-IECO time point. Two-sided *p*-values < 0.05 were considered statistically significant. SAS Version 9.4 (SAS Institute Inc., Cary, NC, United States) was utilized for all analyses.

## Results

### Baseline Characteristics

Table 1 shows the baseline demographic which includes age, sex, race and ethnicity, educational qualifications, employment status, and COVID-19 exposure. The sample is predominantly white (51.2%) and female, with 108 of the 164 patients or 65.85% representing the female sex. The majority of the subjects reported having a bachelor’s or a graduate degree (cumulative percentage ∼62.2%) and employed full-time (51.83%).

**TABLE 1:** Demographic characteristics.

Variable, No. (%)	Baseline population (*n* = 164)
Gender, No. (%)	
Female	108 (65.85)
Age (years), No. (%)	
18 – 29	17 (10.37)
30 – 39	32 (19.51)
40 – 49	36 (21.95)
50 – 59	41 (25)
60 +	36 (21.95)
Race, No. (%)	
White	84 (51.22)
Black or African American	3 (1.83)
Asian	43 (26.22)
Native Hawaiian or Other Pacific Islander	1 (0.61)
American Indian or Alaskan Native	14 (8.54)
Multi-Racial	16 (9.76)
Ethnicity, No. (%)	
Not hispanic/latino	117 (71.34)
Educational qualifications, No. (%)	
High school/GED	13 (7.93)
Some college, associate degree	34 (20.73)
Undergraduate degree (Bachelor’s)	53 (32.32)
Graduate degree (Master’s)	49 (29.88)
Doctoral degree (Ph.D. or equivalent degree)	15 (9.15)
Employment status, No. (%)	
Unemployed	35 (21.34)
Employed: part-time	33 (20.12)
Employed: full-time	85 (51.83)
COVID-19 exposure, No. (%)	
Negative COVID-19 exposure	148 (90.8)

### Primary Outcome–Perceived Stress Score

Shapiro-Wilk test for the difference in the PSS scores showed significant results (*p* = 0.048, < 0.001) indicating non-normality, thus we performed the non-parametric signed-rank test to determine the significant change in median PSS scores for each period.

**FIGURE 2:**
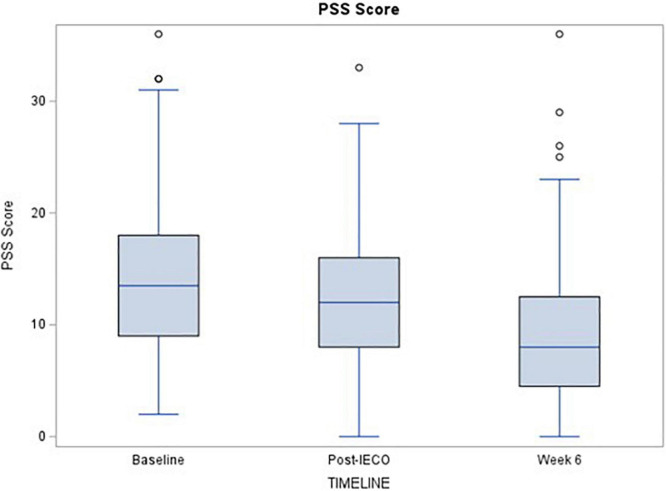
Time trend comparison of PSS scores at all three timepoints. The figure demonstrates the time trend comparison of PSS scores (primary outcome) in complaint participants (*n* = 68) over three timepoints. Wilcoxon ranked sum test was performed to test for significant change in the scores before and after the Inner Engineering Completion Online program. Median (IQR) and the p-values for each comparison are displayed for all timepoints.

The median PSS score for the cohort was significantly lower at all time points. At Post-IECO, median PSS scores for compliant participants (*n* = 95) was 1.5 units less than at baseline (*p* = 0.0023). At 6 weeks, the median PSS score for compliant participants (*n* = 68) was reduced by 3.5 units compared to the post-IECO score (*p* < 0.0001). All of the pairwise difference is statistically significant after the Bonferoni adjustment (Table 2A and Figure 2).

**TABLE 2A:** PSS Scores – primary outcome (primary outcome comparison at three timepoints).

	Baseline vs Post-IECO			Post-IECO vs. Week 6			Baseline vs. Week 6		
	Baseline	Post-IECO	*P*-Value	Post-IECO	Week 6	*P*-Value	Baseline	Week 6	*P*-Value
	(*n* = 95)	(*n* = 95)		(*n* = 68)	(*n* = 68)		(*n* = 68)	(*n* = 68)	
**PSS**	13	12 (8, 16)	0.19	11.5	8	<0.000*	12	8	<0.0001
**Score, median (IQR)**	(9, 18)			(8, 15.5)	(4.5, 12.5)		(8,16)	(4.5, 12.5)	

*Bonferroni adjusted p-value.

*The number of participants assessed is displayed for each outcome as it varies over time due to withdrawals. Median (IQR) and the p-values for each comparison are displayed for all scores by Wilcoxon Rank Sum test with Bonferroni adjustment.*

Furthermore, generalized estimated equations (GEE) analysis was performed to observe the effect of time in predicting PSS scores (Table 2B). Risk ratios are displayed for the time effect with Baseline as the reference. After adjusting for demographics—gender, age, race, ethnicity and educational qualifications, a significant time effect was demonstrated (*p* < 0.0001) with decreasing PSS over time (11% reduction in effect size at post-IECO; 29% reduction in effect size at week six compared to Baseline as reference).

**TABLE 2B:** PSS Score – adjusted and unadjusted model.

	Unadjusted model		Adjusted model	
	RR (95 % CI)	*P*-Value	RR (95 % CI)	*P*-Value
**Timepoint**				
Baseline	Ref.	—–	Ref.	—–
Post – IECO	0.89 (0.83 – 0.95)	0.0005	0.89 (0.83 – 0.96)	0.0015*
Week 6	0.71 (0.63 - 0.80)	< 0.0001	0.71 (0.63 - 0.81)	< 0.0001*

*Significant at Alpha = 0.05.

### Secondary Outcomes

For our secondary outcomes—PSQI, MAAS, and PERMA, we performed the Signed Rank test to determine if there was a significant change between all-time points (Table 3A).

**TABLE 3A:** Comparison of secondary outcomes (PSQI, MAAS and PERMA) at three timepoints.

	Baseline vs. Post-IECO			Post-IECO vs. Week 6			Baseline vs. Week 6		
	Baseline	Post IECO	*P*-Value	Post IECO	Week 6	*P*-Value	Baseline	Week 6	*P*-Value
	(*n* = 94)	(*n* = 94)		(*n* = 68)	(n = 68)		(*n* = 68)	(*n* = 68)	
**Mindfulness score^a^**									
MAAS score,	3.8	4.2	0.001*	4.1	4.4	0.004	3.9	4.4	< 0.001*
Median (IQR)	(2.6, 5)	(3, 5.3)		(2.7, 5.1)	(3.6, 5.1)	5*	(3, 5)	(3.6, 5.1)	
**Sleep scale^b^**									
Global PSQI score,	5	5	0.0094	5	4	0.055	5	4	0.0061*
Median (IQR)	(4, 7)	(4, 7)	*	(4, 7)	(3, 6)		(4, 7)	(3, 6)	
**PERMA scale, negative affect measures^c^**									
Negative emotion,	2.67	1.34	0.0008	2.34	1.34	0.000	2.67	1.34	< 0.0001
Median (IQR)	(1.34, 5)	(1, 2.5)	*	(1, 4.34)	(1, 2.5)	6*	(1.5, 4.67)	(1, 2.5)	*
Loneliness, median	1	1	0.024*	1	1	0.45	1.5	1	0.0001*
(IQR)	(0, 5)	(0,2)		(0,2)	(0,2)		(0, 5.5)	(0,2)	
**PERMA scale, positive affect measures^d^**									
Positive emotion,	7.67	8.17	0.437	8	8.17	0.000	7.67	8.17	0.0002*
Median (IQR)	(6,8.67)	(7.34, 9)		(6.5,8.67)	(7.34, 9)	2*	(6.34, 8.67)	(7.34, 9)	
Engagement, median (IQR)	7.67	8	0.87	8	8	0.03*	8.67	8	0.016*
	(6.34, 8.67)	(7, 8.67)		(6.5, 8.67)	(7, 8.67)		(7.67, 9.67)	(7, 8.67)	
Relationships, median (IQR)	8	8.5	0.018*	8.17	8.5	< 0.00	7.67	8.5	< 0.0001
	(6.34, 9)	(7.17, 9.67)		(6.67, 9.34)	(7.17, 9.67)	01*	(6.6, 8.67)	(7.17, 9.67)	*
Meaning, median (IQR)	8	8.17	0.033*	8	8.17	0.016	8	8.17	< 0.0001
	(6, 9)	(7.34, 9.34)		(6.5, 9)	(7.34, 9.34)	*	(6.34, 9)	(7.34, 9.34)	*
Accomplishment, median (IQR)	7.34	8.17	0.64	7.67	8.17	0.004	7.67	8.17	0.0012*
	(6.34, 8.34)	(7, 9)		(6.5, 9)	(7, 9)	2*	(6.84, 8.34)	(7, 9)	
Overall well-being, median (IQR)	7.62	8	0.17	7.84	8	< 0.00	7.87	8	< 0.0001
	(6.37, 8.56)	(7.5, 8.75)		(6.81, 8.56)	(7.5, 8.75)	01*	(6.6, 8.56)	(7.5, 8.75)	*
Health, Median (IQR)	7.67	8.34	0.036*	8.1	8.34	0.001	8	8.34	< 0.0001
	(6, 9)	(7.67, 9.34)		(7.34, 9)	(7.67, 9.34)	*	(6.83, 9)	(7.67, 9.34)	*

*Significant at Alpha = 0.05.

*^a^Decline in scores suggests successful impact of meditation practices.*

*^b^Increase in scores suggests successful impact of meditation practices.*

*^c^Decline in scores suggests successful impact of meditation practices.*

*^d^Increase in scores suggests successful impact of meditation practices.*

*The number of participants assessed is displayed for each outcome as it varies over time due to withdrawals. Median (IQR) and the p-values for each comparison are displayed for all scores.*

#### Sleep Quality Scale

**FIGURE 3:**
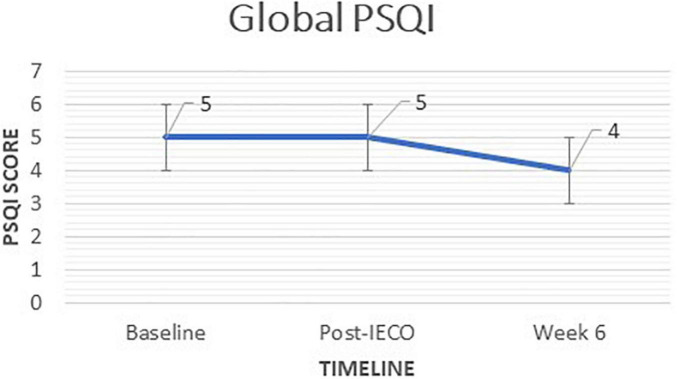
Time trend comparison of Global PSQI scores at all timepoints. The figure demonstrates the time trend comparison of PSQI scores (secondary outcome) in complaint participants (*n* = 68) over three timepoints. Wilcoxon Rank Sum Test was performed to test for significant change in the scores before and after the Inner Engineering Completion Online program. Median (IQR) and the *p*-values for each comparison are displayed for all timepoints.

There was no decrease in global PSQI score, between post-IECO [5.0 (IQR 4–7)] and baseline [5.0 (IQR 4–7)] and between week six follow-up [4.0 (IQR 3–6)] and post-IECO [5.0 (IQR 4–7)]. However, comparing baseline [5.0 (IQR 4–7)] to 6 weeks [4.0 (IQR 3–6)] resulted in 1 point decrease with a *p-*value = 0.0061 which indicates a decline in sleep problems and improvement in sleep quality over time (Figure 3).

#### Mindfulness Scale

**FIGURE 4:**
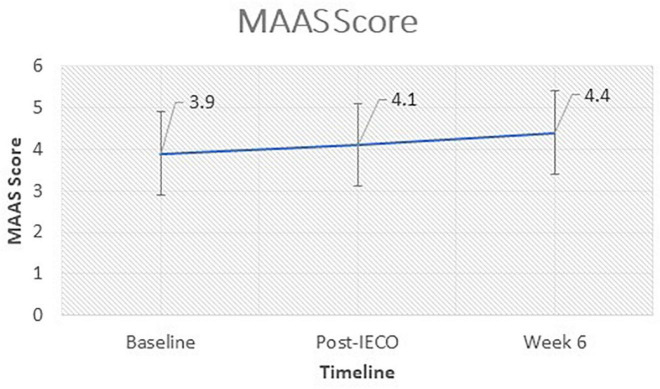
Time trend comparison of MAAS scores at all timepoints. The figure demonstrates the time trend comparison of MAAS scores (secondary outcome) in complaint participants (*n* = 68) over three timepoints. Wilcoxon Rank Sum Test was performed to test for significant change in the scores before and after the Inner Engineering Completion Online program. Median (IQR) and the *p*-values for each comparison are displayed for all timepoints.

The difference in MAAS score between post-IECO [4.2 (IQR 3–5.3)] and Baseline [3.8 (IQR 2.6–5)] was a 0.4 unit increase which was significant (*p* = 0.001) at the 0.05 level. Similarly, the difference between week six follow-up [4.4 (IQR 3.6–5.1)] and post-IECO [4.1 (IQR 2.7–5.1)] was a 0.3 unit increase which was significant (*p* = 0.0045) at the 0.05 level. This indicates an improvement in mindfulness over time (Figure 4).

#### Positive Emotion/Relationship/Engagement Scale

**FIGURE 5:**
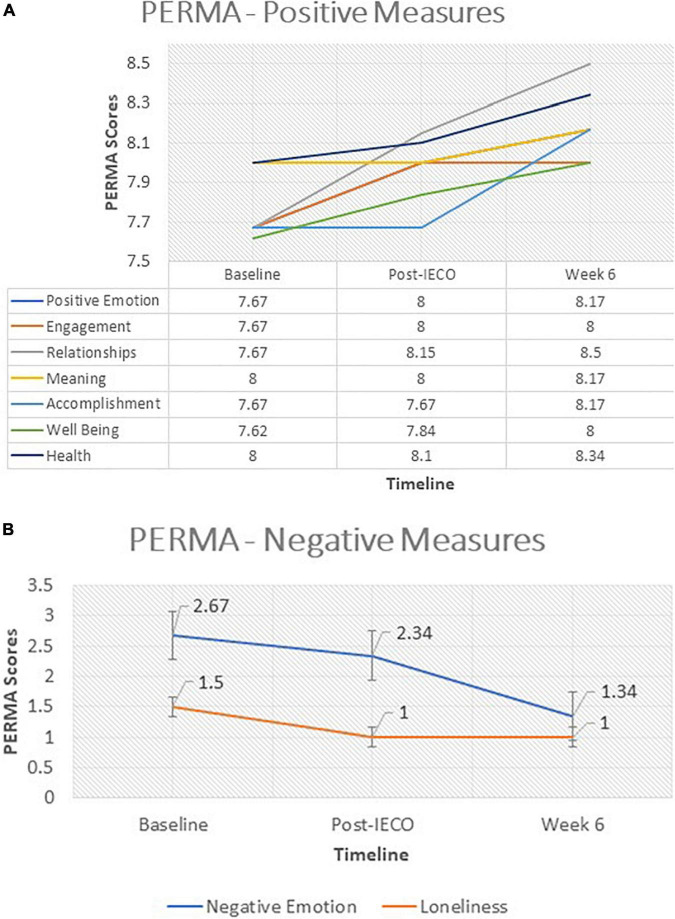
**(A)** Time trend comparison of PERMA scores (positive measures) at all timepoints. The figure demonstrates the time trend comparison of PERMA scores (positive measures) in complaint participants (*n* = 68) over three timepoints. Wilcoxon Rank Sum Test was performed to test for significant change in the scores before and after the Inner Engineering Completion Online program. Median (IQR) and the *p*-values for each comparison are displayed for all timepoints. **(B)** Time trend comparison of PERMA scores (negative measures) at all timepoints. The figure demonstrates the time trend comparison of PERMA scores (negative measures) in complaint participants (*n* = 68) over three timepoints. Wilcoxon Rank Sum Test was performed to test for significant change in the scores before and after the Inner Engineering Completion Online program. Median (IQR) and the p-values for each comparison are displayed for all timepoints.

Positive emotion/relationship/engagement scale components consist of both negative and positive effects. The median difference in all positive PERMA components was about 0.5–0.1 unit increase, some of which were significant at the 0.05 level across all time points [(Relationships: (*p* = 0.018)–Baseline [8 (IQR 6.34–9)]; Post-IECO [8.5 (IQR 7.17–9.67)] | (*p* < 0.0001)–Post-IECO [8.17 (IQR 6.67–9.34)]; week six [8.5 (IQR 7.17–9.67)]); (Meaning: (*p* = 0.033)–Baseline [8 (IQR 6–9)]; Post-IECO [8.17 (IQR 7.34–9.34)] | (*p* = 0.016)–Post-IECO [8 (IQR 6.5–9)]; week six [8.17 (IQR 7.34–9.34)]); and (Health: (*p* = 0.036)–Baseline [7.67 (IQR 6–9)]; Post-IECO [8.34 (IQR 7.67–9.34)] | (*p* = 0.001)–Post-IECO [8.1 (IQR 7.34–9)]; week six [8.34 (IQR 7.67–9.34)])] (Figure 5A).

Similarly, for all negative PERMA effects, there was about 0.5–1 unit decrease which was significant at the 0.05 level across all time points (Negative Emotion: (*p* = 0.0008)–Baseline [2.67 (IQR 1.34–5)]; post-IECO [1.34 (IQR 1–2.5)] | (*p* = 0.006)–post-IECO [2.34 (IQR 1–4.34)]; week six [1.34 (IQR 1–2.5)]). This indicates a significant decline of negative emotions and improvement in positive emotions over time (Figure 5B).

Next, GEE analysis was performed to observe the effect of time in predicting the secondary outcomes PSQI and MAAS (Table 3B). Risk ratios are displayed for the time effect with Baseline as the reference. After adjusting for demographics (gender, age, race, ethnicity, country of residence, and educational qualifications), a significant time effect was demonstrated (*p* < 0.0001) with increasing MAAS over time, likely due to learning effects (8% increase in effect size at post-IECO; 15% increase in effect size at week six compared to Baseline as reference). A similar significant time effect was observed for Global PSQI scores.

**TABLE 3B:** Secondary outcomes (MAAS and Global PSQI) using GEE modeling to observe the effect of time on secondary outcomes.

Scores	Baseline	Post-IECO	6 Weeks	*P*-Value
**MAAS score**				
Number assessed	164	95	68	
Median (IQR)	3.8 (2.6, 5)	4.2 (3, 5.2)	4.4 (3.6, 5.1)	
Time effect	Ref.	RR = 1.08	RR = 1.15	< 0.0001*
**Global PSQI score**				
Number assessed	164	95	68	
Median (IQR)	6 (4, 8)	5 (4, 7)	4 (3, 6)	
Time effect	Ref.	RR = 0.88	RR = 0.82	0.0006

*Significant at Alpha = 0.05.

#### Correlation Between the Primary and Secondary Outcomes

Spearman correlational analyses between the primary outcome change and secondary outcome changes between baseline and 6th week showed significant results (*p* < 0.05) for all categories except PSQI. PSQI change was weakly correlated to the PSS change (*r* = 0.16), though not significant at 0.05 level. MASS change had weakly positive correlation to the PSS change (*r* = 0.39). All PERMA Positive Affect Measures [positive emotions (*r* = –0.59), engagement (*r* = –0.42), relationships (*r* = –0.28), meaning (*r* = –0.43), accomplishment (*r* = –0.52), overall (*r* = –0.56)] had negative correlations indicating successful impact of meditation practice: PSS decreased, and positive affect measures increased. All PERMA Negative Affect Measures [negative emotions (*r* = 0.58), loneliness (*r* = 0.30)] had positive correlation indicates successful impact of meditation practice: Both PSS and negative affect measures decreased. The correlational analyses above showed co-fluctuations of the outcomes throughout the meditation sessions (Table 4).

**TABLE 4:** Correlation of primary and secondary outcomes (change of baseline vs week six).

Spearman correlation (*n* = 68) r (*p*-value)	PSS
PSQI	0.16 (p = 0.19)
MAAS	0.39 (p< 0.01)*
PERMA positive emotion	−0.59 (p< 0.01)*^a^
PERMA engagement	−0.42 (p< 0.01)*
PERMA relationships	−0.28 (p = 0.02)*
PERMA meaning	−0.43 (p< 0.01)*
PERMA accomplish	−0.52 (p< 0.01)*
PERMA overall well-being	−0.56 (p< 0.01)*
PERMA health	−0.27 (p = 0.02)*
PERMA negative	0.58 (p <0.01)*^b^
PERMA loneliness	0.30 (p = 0.01)*

*Significant at Alpha = 0.05.

*^a^Negative correlation indicates successful impact of meditation practice: i.e.: PSS decreased, and positive affect measures increased.*

*^b^Positive correlation indicates successful impact of meditation practice: Both PSS and negative affect measures decreased.*

### Compliance

Of the 375 participants who were interested in participating in this study, we excluded participants who did not meet our inclusion criteria (*n* = 64); did not give their consent for participating in the study (*n* = 111); had missing demographic data (*n* = 26) and/or Perceived Stress Score data (*n* = 10), resulting in a final sample size of 164. Over the follow-up period for this prospective cohort study, the compliant participant pool has decreased by 30–40% at each time point (Baseline—*n* = 164; post IECO—*n* = 95 and week six—*n* = 68).

To accommodate for survival bias, an exploratory analysis was also done to measure the progress of those participants who dropped off from the study at the post-IECO time point (*n* = 23). Significant change was observed in PSS, MAAS, and PSQI scores resulting in decreased stress, improved sleep quality and mindfulness, merely following initiation into the practices (Refer to Supplementary Tables A, B).

## Discussion

This study aimed to investigate the effect of a remotely delivered, online meditation workshop, IECO, on several aspects of well-being, including perceived stress, sleep quality, and both positive and negative dimensions of flourishing. Participants of the IECO conference were assessed on the Perceived Stress Scale (PSS), PSQI, the PERMA Profiler, and the MAAS.

### Stress Reduction

As expected, PSS scores decreased from Baseline to immediately post-IECO and from Baseline to the 6-weeks follow-up (refer to Table 2A). There was a significant time effect to this decrease, suggesting that the observed effect increases with increased duration of practice. Both decreases in PSS scores remained significant in the adjusted model (refer to Figure 2 and Table 2B).

Among the mind body intervention (MBI’s), MBSR has demonstrated strong evidence in reducing stress in healthy adult population (Bartlett et al., 2019). In traditional MBSR, participants learn different methods of meditation (body scan, awareness of breathing sensations, loving-kindness) and mindful movements (modified Hatha yoga and other bodywork) while sharing their experiences in a group setting. Typically, MBSR program spans 8 weeks and includes weekly 2.5-h classes, a day-long retreat, and prescribed homework and daily practices with audio guide, including a minimum of 40 min of meditation. Participants are free to choose any intervention they wish to pursue over time. IECO on the contrary offers a more structured approach to its novice participants. Following initiation into the practices, participants are asked to repeat a set of practices including pranayama (breath work), aum chanting and meditation twice daily. Each session last for 21 min at least and participants are advised to practice the intervention routinely for a at least 6 weeks. Our study demonstrated that IECO practices resulted in a significant reduction in perceived stress despite online delivery of the intervention and a lack of rigor (e.g., weekly classes, prescribed homework) noted in traditional MBSR delivery.

Next, it has been noted that vacations have an ameliorating effect on stress, and that this may be a part of the positive effects of meditation retreats (Epel et al., 2016; May et al., 2020; Blasche et al., 2021). Vacations result in positive health effects by reducing exhaustion and improving satisfaction. However, these changes are not permanent and fade out upon resumption of routine life (de Bloom et al., 2009). Gilbert et al. (2014) conducted a randomized controlled trial to compare the changes in stress and wellbeing reported by non-meditators attending a yoga retreat vs. vacationing in the same resort. They noted that although both groups reported a decline in stress and improvement in overall well-being, only retreat participants showed decreased rumination and better control over stressors (Gilbert et al., 2014).

The vacation effect’ is unlikely to be the primary driver of the reduction in perceived stress in this study, given it is short-term (Blasche et al., 2021) and because this program was conducted online amid a pandemic. Therefore, although there have been studies on the effect of this program on perceived stress before (Peterson et al., 2017), the present study is the first to our knowledge that possibly accounts for the “vacation effect.” Overall, these results suggest that the IECO program is associated with a reduction in perceived stress, a finding in line with the literature on meditation (Lee et al., 2007; Goyal et al., 2014; Khalsa, 2015; Edwards et al., 2018).

### Mindfulness

Analogous to the cognitive reappraisal techniques taught in MBSR, IEO which is a pre-requisite for IECO requires its participants to employ logic-based self-inquiry. This investigation of everyday human experiences is often accompanied by humorous wisdom-based stories (Sadhguru, 2016). Gaining the ability to reappraise one’s internal mental and physiological processes and external situations and social relations one encounters reduced autonomic reactivity to internal and external stressors.

Mindful attention awareness scale scores increased at all time points (refer to Figure 4), suggesting increased mindfulness over time. Mindfulness is described as a heightened state of non-judgmental awareness of one’s mental activity and other sensory experiences. A state of mindfulness has been associated with greater feelings of well-being possibly due to the predominance of sense experience over rumination when it is achieved. MAAS scores suggest higher levels of mindfulness levels correlate with lower levels of the neuroticism personality trait, and are inversely correlated with depression, self-consciousness and angry hostility (Brown and Ryan, 2003). Therefore, increasing their levels of mindfulness may be a means for the participants to better self-regulate internal states by increasing non-judgmental awareness and reducing rumination.

### Increasing Well-Being

Positive emotion/relationship/engagement scale Profile scores for negative affect decreased (refer to Figure 5B) and those for positive affect increased over each time point (refer to Figure 5A). This is in line with several studies reported in the literature (Khalsa, 2015; Cavanagh et al., 2018; Edwards et al., 2018; Allen et al., 2021).

Unfortunately, disproportionate amount of focus is applied to dysfunctional psychiatric issues such as anxiety and depression while, positive psychological outcomes such as emotional intelligence, positive emotions, vitality, flourishing, and life satisfaction and work-related outcomes such as job performance and work engagement await more research (Lomas et al., 2019). Among the MBI’s, only interventions that focus on Loving-Kindness Meditation (LKM) have demonstrated a medium effect size on daily positive emotion (Zeng et al., 2015).

Interestingly, a meta-analysis of meditation studies found weak to moderate evidence for the reducing stress after meditation but did not find any effect on positive affect or well-being (Goyal et al., 2014). The difference may be accounted for because the meta-analysis only included participants with a diagnosed clinical condition. It may be challenging to improve positive affect due to their existing ill-health.

### Sleep Quality

Finally, subjects reported better subjective sleep quality over time (refer to Figure 3). However, the difference in sleep quality was only statistically significant between Baseline and post-IECO and baseline and the week-six follow up (refer to Table 3A). Perhaps a floor effect may account for the lack of a significant difference between the post-IECO, and week-six follow up time points. Furthermore, there was no significant time effect to this decrease in the adjusted model (refer to Table 3B). This is in accordance with the current literature (Rusch et al., 2019). One randomized control trial found, for example, that older adults assigned to a mindful awareness practice showed more significant improvements on the PSQI than those assigned to sleep hygiene education (Black et al., 2015). These subjective reports were validated by polysomnography in another randomized control trial (Guerra et al., 2020).

### Intervention Delivery Method

Various flexible delivery methods have been experimented, ranging from blended learning (classroom meeting combined with online practices or e-coaching) to completely online, videoconferencing, or audio tracks. However, most MBIs are still taught in face-to-face group format (Bartlett et al., 2019). Vonderlin et al. (2020) observe that 79% of the programs (79%) are delivered in-person, followed by online programs (13%), combinations of online and in-class elements (7%), or via audio records (1%). A recent review of online MBSR in non-work settings found equivalent effects of online to those of face-to-face class-based training (Lomas et al., 2019). As previously mentioned, this is the first time IECO is being offered in an online format and therefore, this study adds to the growing literature on meditation by providing evidence that, even when taught online, meditation is associated with decreased perceived stress, improved subjective sleep quality and better outcomes in various aspects of well-being, including health, engagement, and relationships. However, flexible delivery modes, such as online or app, have been under-investigated and presents an opportunity for research to examine the effectiveness of online or app-based program in greater details.

### Strengths, Limitations, and Future Directions

The strengths of this study lie in its assessment of a wide range of measures of well-being, its longitudinal design, which allowed for a time-effect analysis, and in type of intervention (remote), which allowed for an accounting of the vacation effect.

The primary limitation of this study is its observational design. It has been noted that studies without randomization or active control are subject to biases and self-selection (Chambless and Hollon, 1998). Individuals who exert effort or expend time and resources to join a meditation retreat believe that meditation will positively affect them. When studies do not include objective measurements, self-reports can be colored by personal biases. To promote objective assessment in our study, validated neuropsychological scales were employed as outcome measures at the study design phase. Because of the ongoing pandemic, we could not verify subject compliance with objective, physiological measures and thus had to rely on the subjects’ self-reports of compliance. Statistical analysis employing repeated observations in the same individuals over a six-week time frame was intended to improve data analysis rigor of the self-reports and mitigate the lack of randomization and control group.

Another limitation was participant attrition (30–40% at each time point and 59% from Baseline to the week six follow-up.) This is in line with the other mindfulness studies, which had attrition rates ranging from 8–60% (Spijkerman et al., 2016; Cavanagh et al., 2018). An analysis of the primary and secondary outcomes of those participants that left the study between the post-IECO and week six follow up time points was conducted to see if they experienced changes between the baseline and post-IECO timepoints. Participants still experienced significant decreases in perceived stress and increases in mindfulness and sleep quality (Supplementary Tables A, B). Conversely, none of the PERMA scale sub-sections showed a significant change, except for Meaning and Health. These results suggest that study participants did not drop out of the study because they believed the intervention had no effect, and that both positive and negative emotions, as well as the other positive effects, may require more time to change than perceived stress. Future studies should attempt to control attrition bias by incentivizing participants to complete study protocols or including weekly refresher interventions.

Future studies should focus on a robust design—ideally a randomized control trial—and the mechanisms of action for the beneficial effects of this style of meditation. Focusing on inflammatory mediators, cortisol, or epinephrine and norepinephrine may provide supporting evidence to participants’ self-reports. Other means of investigating the mechanism of action would be by looking at heart rate variability, which is a measure of sympathetic nervous system activation and could be affected by meditation (Libby et al., 2012; Muralikrishnan et al., 2012; Koerten et al., 2020) or the anterior cingulate cortex and ventromedial prefrontal cortex, the activation of which is associated with meditation-related reduction of anxiety (Zeidan et al., 2014). An exciting direction for meditation research would be to investigate the mechanism by which meditation may affect sleep architecture and quality (Maruthai et al., 2016).

Furthermore, the Inner Engineering Online program contains sessions of intellectual discussions on how to frame life events and stressors, be “joyful by your own nature,” and 21-min meditation practice to be performed daily. This meditation practice can be broken down into individual steps of breath regulation, mantra chanting, and focused attention meditation. Future studies on this practice should investigate whether these steps have unique effects.

## Conclusion

This study provides significant evidence that, mind-body interventions, such as Shambhavi Mahamudra Kriya, when taught online and practiced for just 6 weeks are associated with decreased perceived stress, improved subjective sleep quality, and better outcomes in various aspects of well-being, including health, engagement, and relationships.

## Data Availability Statement

The raw data supporting the conclusions of this article will be made available by the authors, without undue reservation.

## Ethics Statement

The studies involving human participants were reviewed and approved by Committee on Clinical Investigations at Beth Israel Deaconess Medical Center. The patients/participants provided their written informed consent to participate in this study.

## Author Contributions

BS, SH, and KK conceptualized the study’s design. PU, AJ, and IM conducted the data collection. PU, IM, LN, and LK carried out the data analysis. PU did project administration and supervision. LN, IM, and LK verified the underlying data. All authors contributed to the writing, review, and editing, confirmed that they had full access to all the data in the study, and accepted the responsibility to submit for publication.

## Conflict of Interest

The authors declare that the research was conducted in the absence of any commercial or financial relationships that could be construed as a potential conflict of interest.

## Publisher’s Note

All claims expressed in this article are solely those of the authors and do not necessarily represent those of their affiliated organizations, or those of the publisher, the editors and the reviewers. Any product that may be evaluated in this article, or claim that may be made by its manufacturer, is not guaranteed or endorsed by the publisher.
