# Supplementary material for: Short Term Effects of Inner Engineering Completion Online Program on Stress and Well-Being Measures
Source: Front Psychol. 2022 Apr 27;13:814224. doi: 10.3389/fpsyg.2022.814224 (PMC9094408; doi:10.3389/fpsyg.2022.814224)
Supplement: Supplementary file 1 [file Data_Sheet_1.docx]

**Supplementary material**

***Table A: Primary Endpoint: PSS Score for people who left the study at post-IECO***

|  | Baseline vs Post-IECO | | |
| --- | --- | --- | --- |
|  | Baseline (n=27) | Post-IECO (n=27) | P-Value |
| PSS Score, Median (IQR) | 16 (12, 20) | 13 (7,17) | 0.011* |

** Significant at Alpha = 0.05*

***Table B: Secondary Endpoint: MAAS, PSQI and PERMA Score for people who left the study at post-IECO***

|  | Baseline vs Post-IECO | | |
| --- | --- | --- | --- |
|  | Baseline (n=27) | Post-IECO (n=27) | P-Value |
| Mindfulness Score |  |  |  |
| MAAS Score, Median (IQR) | 3.4 (2.2, 5) | 4.4 (3.8, 5.6) | 0.006* |
| Sleep Scale |  |  |  |
| Global PSQI Score, Median (IQR) | 6 (4, 8) | 4 (4, 8) | 0.062 |
| PERMA Scale, Negative Affects ^d^ |  |  |  |
| Negative Emotion, Median (IQR) | 3.34 (1.34, 5) | 2.5 (1.34, 3.67) | 0.08 |
| Loneliness, Median (IQR) | 1 (1, 5) | 1.5 (1,4) | 0.65 |
| PERMA Scale, Positive Affects ^d^ |  |  |  |
| Positive Emotion, Median (IQR) | 7.34(5,8.34) | 7.67 (6.34, 8.34) | 0.53 |
| Engagement, Median (IQR) | 7 (6.34, 8) | 7.34 (6, 8.34) | 0.85 |
| Relationships, Median (IQR) | 7.34 (6.34, 8.34) | 7.67 (6.67, 8.34) | 0.78 |
| Meaning, Median (IQR) | 7 (5.34, 8) | 7.67 (7, 8.67) | 0.016* |
| Accomplishment, Median (IQR) | 7.34 (5.67, 8.34) | 7.31 (6.87, 8.18) | 0.82 |
| Overall Well-Being, Median (IQR) | 7.25 (5.81, 8.56) | 8 (7.5, 8.75) | 0.53 |
| Health, Median (IQR) | 6.67 (5.34, 8.34) | 7 (6, 9) | 0.049* |

** Significant at Alpha = 0.05*

*^a^ Decline in scores suggests successful impact of meditation practices*

*^b^ Decline in scores suggests successful impact of meditation practices*

*^c^ Decline in scores suggests successful impact of meditation practices*

*^d^ Increase in scores suggests successful impact of meditation practices*
